# Integrating navigation assistance for redirecting freehanded spinal instrumentation: experience and technique

**DOI:** 10.1007/s11701-023-01686-9

**Published:** 2023-09-04

**Authors:** Lauren Stone, Marin McDonald, Luke Wojdyla, Joseph A. Osorio

**Affiliations:** 1 Department of Neurological Surgery, UC San Diego, San Diego, CA USA; 2 Department of Neuroradiology, UC San Diego, San Diego, CA USA

**Keywords:** Spine, Deformity, Navigation, Robotic

## Abstract

Retrospective review of all spinal fusions > 3 levels involving the thoracolumbar and/or sacroiliac at a single institution, by a single surgeon between 3/12/2020 and 8/13/2021 were reviewed. All screws that were secondarily navigated after identified as misdirected on intraoperative CT scan were included. Neuromonitoring reports were culled for mA threshold to triggered EMG response for all redirected screws. Intraoperative, post-de novo screw placement images (fluoroscopy scout and intraoperative CT) and post-redirection intraoperative scoliosis films and post-operative scoliosis films were independently reviewed by a senior neuroradiologist. Fifty redirected screws in the thoracic, lumbar, sacral, and ilium were identified as misdirected and redirected via navigation. The new trajectory of all screws was confirmed satisfactory by independent review between a senior neuroradiologist and neurosurgeon. Four screws could not be verified by post-operative imaging (4/50, 8%). All triggered EMG stimulated > 15 mA. No screws required return to the operating room for revision. No patients experienced a post-operative deficit. Redirection of misdirected thoracolumbar and sacroiliac screws can be performed using intraoperative CT and navigation as a means to detect and directly visualize appropriate placement.

## Introduction

Pedicle screw and sacroiliac fixation are mainstay techniques in adult deformity spine correction. Accurate bony purchase bears consequence on the final integrity of the individual screw and construct as a whole [1]. For this reason, misdirected screws are routinely redirected. Doing so requires (1) a means to identify erroneous placement and (2) a workflow to replace and test accurate redirection. Today, new intraoperative imaging linked to robotic registration allows for real-time, 3D reconstructed models for stereotactic guidance and live navigation. While several papers have published regarding the accuracy of navigated instrumentation for de novo screws, a discussion regarding the workflow and outcome for secondary redirection for misdirected instrumentation has yet to be discussed [2–6]. In this paper, we review our experience and technique using a navigated, shared-control robotic system as a means to detect and redirect misdirected thoracolumbar and sacroiliac screws.

## Methods

We retrospectively reviewed all spinal fusions > 3 levels involving the thoracolumbar and/or sacroiliac spinal segments at a single institution, by a single surgeon between 3/12/2020 and 8/13/2021. Operative reports were reviewed for (1) level and laterality of identified misdirected screws (2) removal and redirection of misdirected screws (2) intraoperative CT scan and fluoroscopy in the picture archiving and communication system (3) neuromonitoring reports. Reports in which screws were primarily navigated, intraoperative or post-operative imaging was missing, or associated neuromonitoring reports were missing were excluded. Our intraoperative imaging and navigation workflow is discussed below. Intraoperative, post-de novo screw placement images (fluoroscopy scout and intraoperative CT) and post-redirection intraoperative scoliosis films and post-operative scoliosis films were independently reviewed by a senior neuroradiologist. Neuromonitoring reports were culled for mA threshold to triggered EMG response for all redirected screws. Post-operative neurologic exam and any return to the operating room for instrumentation revision was evaluated.

## Navigation workflow and technique

**Fig. 1:**
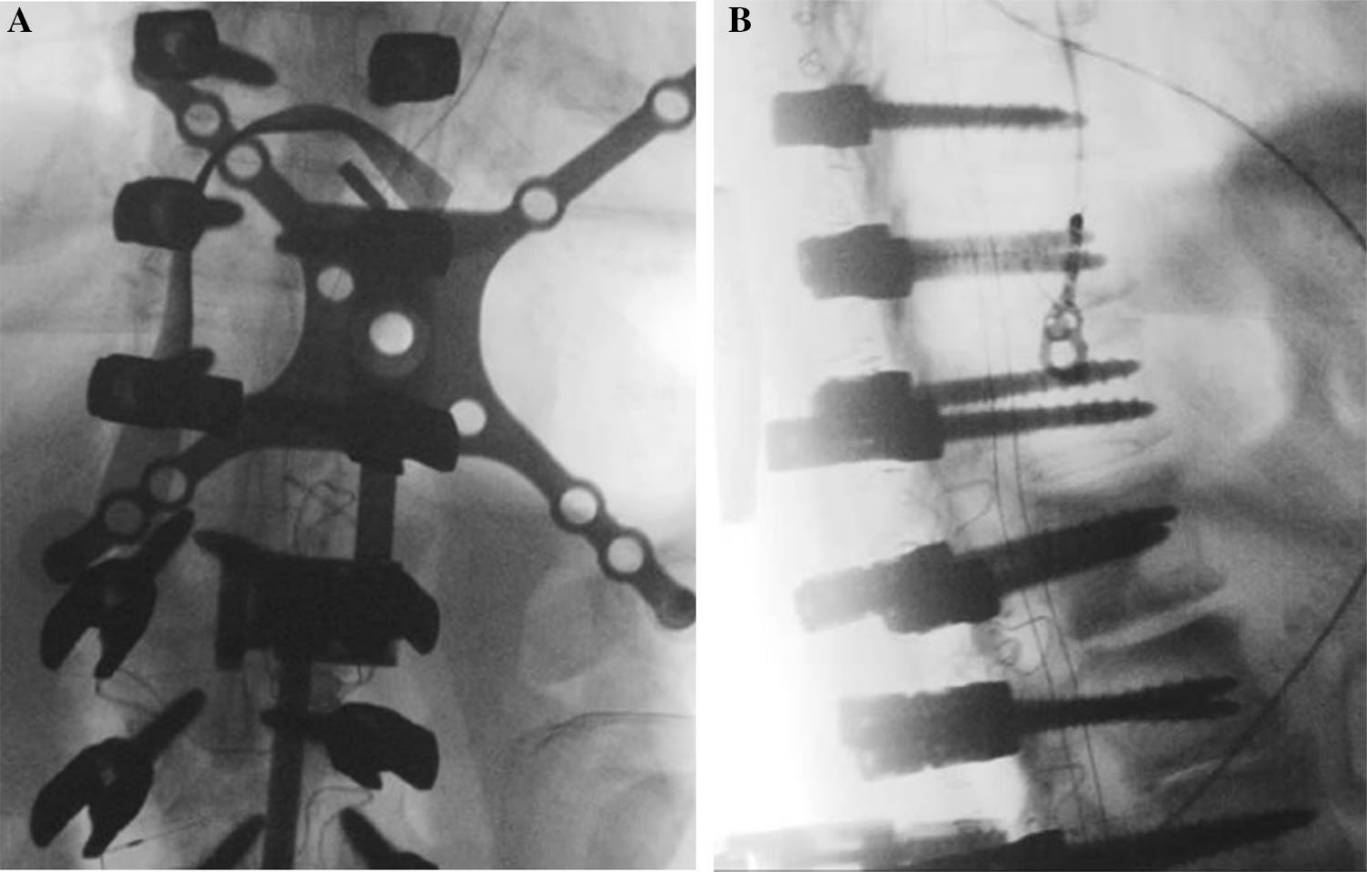
**A**, **B** Intraoperative AP and lateral scout image for CT. Spinous process array is also seen at the inferior of the image

**Fig. 2:**
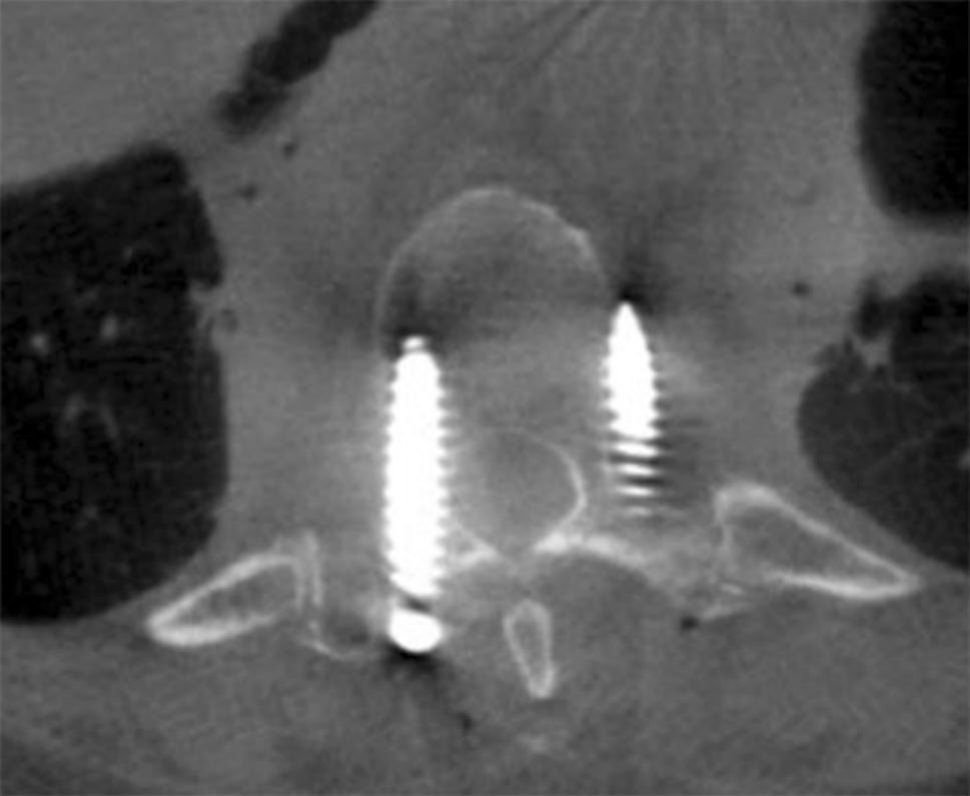
Intraoperative axial CT with lateral breach

The patient is brought to the operating theater and inducted under anesthesia. Gardner-Wells tongs are placed in standard fashion and the patient is transferred prone to a Jackson table. Neuromonitoring leads are placed for triggered, motor-evoked, and somatosensory-evoked potentials in the upper and lower extremities. Incision then proceeds, followed by subperiosteal dissection, and exposure of appropriate landmarks for pedicle, S2A1, and iliac instrumentation is performed. Screws are placed in standard freehand fashion. A spinous process reference probe is then securely fastened to the base of the exposed levels in view of a reference array (Medtronic StealthStation, Medtronic, Minneapolis). The O-Arm (Medtronic, Minneapolis, MN, USA) is then positioned around the patient and a scout image is performed to delineate the region to be scanned. A CT scan is then performed (Fig. 1A, B) The images are uploaded onto a viewing station for axial, sagittal, and coronal evaluation (Fig. 2). Misplaced screws are independently evaluated by a resident and attending neurosurgeon. Screws to be replaced at marked by level, laterality, and redirection trajectory. The CT images are then uploaded to the navigation system for registration.

**Fig. 3:**
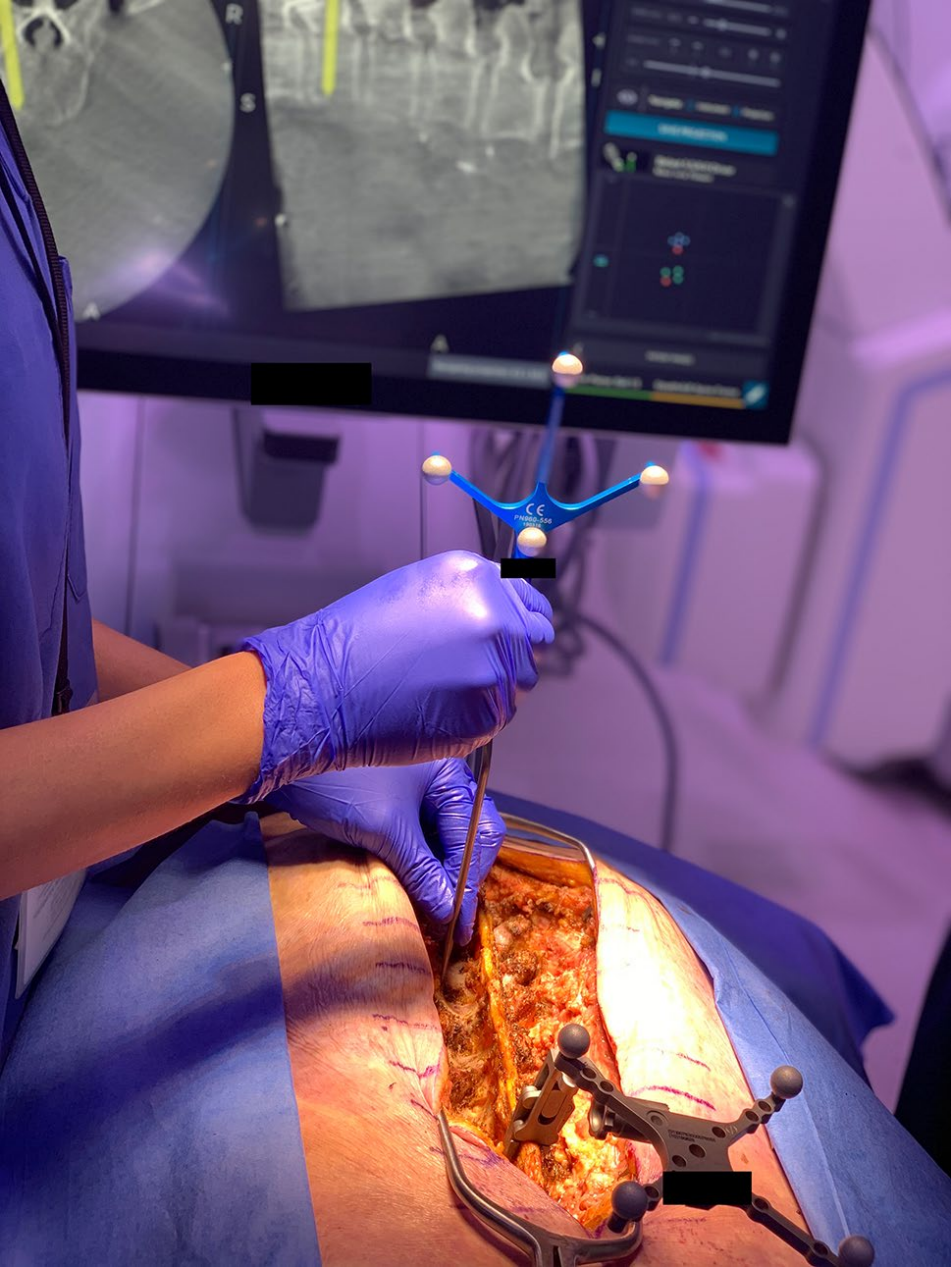
Cadaveric model demonstrating probe and 3D reconstruction showing projected screw entry and length. Reference array is seen clamped to the spinous process

**Fig. 4:**
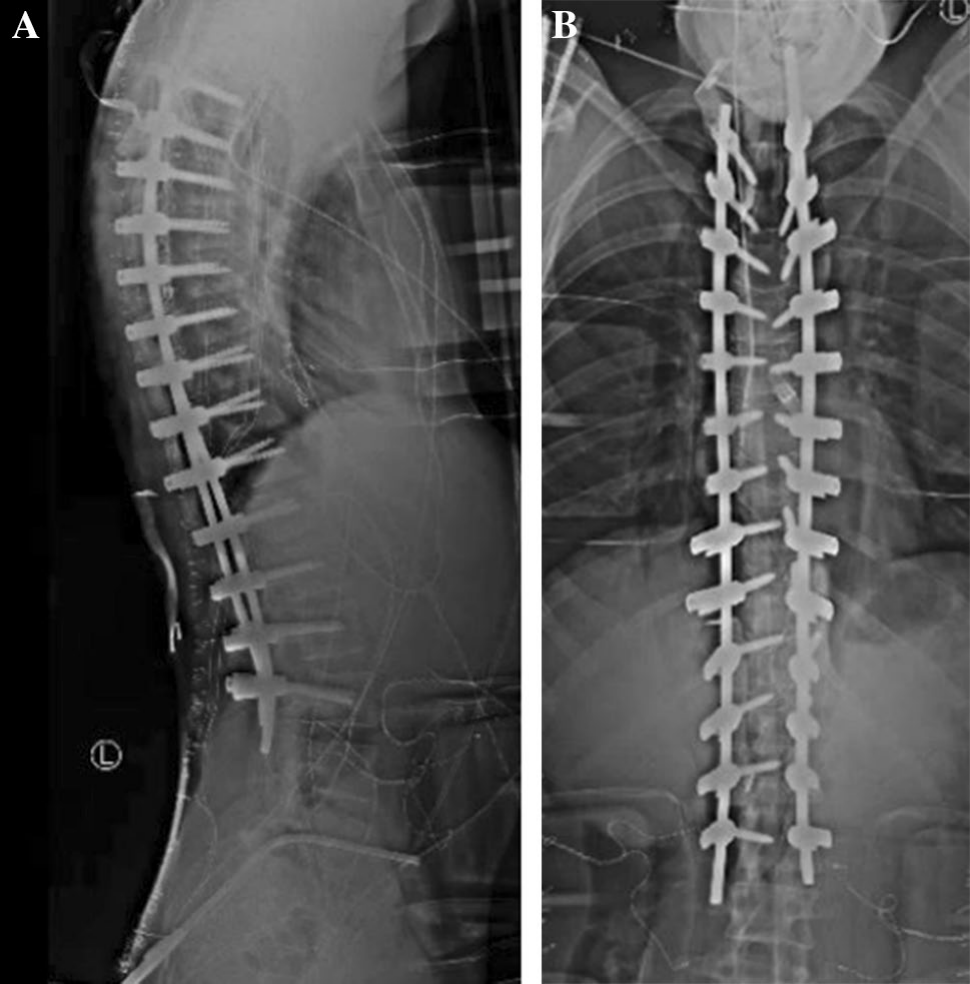
**A**, **B** Intraoperative AP and lateral scoliosis films after final instrumentation and rod placement

The field is re-exposed and the misdirected screw is removed. A tactile probe is used to define the optimal corridor trajectory (Fig. 3). A pilot hole is made with a navigated handheld drill followed by cannulation of the new tract with a pedicle finder. The hole is probed for breach. The appropriate screw diameter and length is selected and uploaded into the navigation software. A navigated screwdriver is used to place the new screw under live visualization into the freshly cannulated corridor. The final turns are then performed manually for tactile feedback. All screws undergo triggered EMG testing to threshold with > 15 mA and evaluated in real-time by a neurologist specialized in intraoperative neuromonitoring. Rods are then contoured and placed in standard fashion. An intraoperative scoliosis film is performed to evaluate general alignment and screw trajectories (Fig. 4A, B). Post-redirection CT spins are not performed due to additional radiation exposure to the patient. Decortication, allograft/autograft, and closure proceed in standard fashion.

## Results

Fifty redirected screws (29 thoracic; 7 lumbar; 12 sacral; 2 iliac) from 19 procedures were retrospectively reviewed in operative reports. No cases with documented navigated, redirected screws were excluded for lack of imaging or intraoperative reports. Intraoperative CT scans were reviewed by an attending neurosurgeon and independently reviewed by a neuroradiologist for agreement in level and laterality of misdirection and post-navigation correction.

Misdirected screws were classified as follows: 13 medial, 18 lateral, 4 inferior, 3 superior, 3 long, 2 inferolateral, 1 superolateral. The new trajectory of four redirected screws could not be satisfactorily evaluated by our neuroradiologist on post-redirection imaging (46/50, 92%). One screw appeared to be misdirected after navigation (45/46, 97.2%; see Table 1). This and all redirected screws stimulated at > 15 mA on triggered EMG. There was no recorded post-operative deficit in patients with redirected screws. No screws required return to OR for redirection. No attempts at navigated screws were aborted due to poor registration or technological error.

**Table 1:** Operative Report Identified and Evaluated Misdirected Screws

**Procedure**	**Laterality**	**Level**	**Misdirection Identified**
T10-Ilium	R	T9	medial
T10-Ilium	R	S1	superolateral
L3-5	L	S1	lateral/superior
T3-Ilium	L	S1	**Not confirmed**
T3-Ilium	R	S2AI	lateral
T3-Ilium	L	T3	medial
T3-Ilium	R	L 4	medial
T3-Ilium	R	L3	medial
T3-Ilium	R	T10	medial
T3-Ilium	R	T3	medial
T3-Ilium	R	T9	medial
C2-L1	R	T11	inferolateral
C2-L1	R	T2	inferolateral
C2-L1	R	T5	medial
T9-Ilium	R	T10	lateral
T9-Ilium	R	T11	lateral
T9-Ilium	R	T12	lateral
L2-ilium	L	L2	medial
T10-Ilium	L	S1	medial
T10-Ilium	R	S1	superior
T10-Pelvis	R	L1	medial
T10-Ilium	L	L1	inferior
T10-Ilium	R	S2	inferior
T10-Ilium	L	T10	lateral
T10-Ilium	R	S1	lateral
T10-Ilium	L	T10	lateral
T10-Ilium	L	S2A1	long
T4-Ilium	L	T4	lateral
T4-Ilium	L	T5	lateral
T4-Ilium	L	Ilium	long
T8-Pelvis	L	T8	lateral
T8-Pelvis	R	T9	lateral
T8-Pelvis	R	T10	medial
T8-Pelvis	L	S1	superior
T10-Ilium	R	S2A1	**Not confirmed**
T9-Ilium	R	L4	lateral
T9-Ilium	R	L3	lateral
T3-L2	R	T3	lateral
T3-L2	R	T6	inferior
T3-L2	R	T2	lateral
T4-Ilium	R	S1	**Not confirmed**
T4-Ilium	R	T4	**Not confirmed**
T4-Ilium	R	Iliac	long/superior
T4-Ilium	L	S1	superior
T1-9	R	T7	inferior
T1-9	L	T6	lateral
C2-T10	R	T8	medial
T3-L3	L	T4	lateral
T3-L3	R	T3	lateral
T10-Ilium	R	T10	lateral

## Discussion

The rationale for redirecting misplaced screws has been explored in a number of thoughtful studies. Brasiliense et al. reported a 66% loss in pull-out strength in de novo “airball” screws (out of vertebral body) with 21% loss for laterally directed screws in their thoracic cadaveric model [7]. These findings have been repeated elsewhere in both the thoracic and lumbar spine [8, 9]. Kothe et al. further evaluated the integrity of a construct as a whole after pedicle breach, finding significant increases in rotational and lateral range of motion in the construct and overall decreases in construct stability [1].

Additional models have evaluated regained pull-out strength with screw redirection. Goda et al. human cadaveric study comparing primarily placed and secondarily redirected pedicle screws found at 24% loss of pull-out strength in the latter after a lateral breach [10]. The percentage varies in the literature with reports from 11 to 71% decrease in pull-out strength in human and bovine cadaveric models in both the thoracic and lumbar spine [11, 12] It should be noted, though, that this higher reported percents exceed the findings for de novo misdirected screws, emphasizing heterogeneity in the literature that warrants caution on interpretation.

Methods of evaluating misplaced screws include probing for cortical breaches, neuromonitoring, and intraoperative imaging with technological adjuncts for live anatomic reconstructions [13]. The latter of these options has evolved from static images to interactive, real-time modeling. In 2018, the Congress of Neurological Surgeons published the white paper on *Robotics in Neurosurgery*, delineating the foreseeable future of robotics in three main areas: telesurgical, supervisory controlled, and shared-control modes. This latter mode includes technologies in which the robot performs controlled functions, typically as a system registered to the patient, but under the direct control of the surgeon who will use the robot’s assistance for a specific goal [14].

Navigated assistance has gained popularity in spine surgery these new systems are coapted with known operative tools (i.e., intraoperative CT, fluoroscopy). Full integration of intraoperative CT assisted screw placement has been reported in large studies with early favorable results, with one prospective, post-marketing registry reporting a 1.8% need for revision in their series of 192 screw series [15]. This proof of concept is augmented by Jin et al. head to head comparison between O-arm and freehand screws in their study of 341 pedicle screw study (90% freehand versus 95.8% O-arm, respectively), although statistical significance was only obtained in the thoracic spine [16].

Other workflows emphasize technology as one of several safety checks for accurate screw placement. For example, Kassis et al. found that an intraoperative CT scan increased the sensitivity for detecting abnormal screw placement when preceded by an abnormal triggered EMG. This approach advocates for complementary, multimodal safety checks for navigated instrumentation [17].

There is currently no standard, generalizable consensus on the overall benefit patient receiving de novo navigated instrumentation versus freehand. Discussion of the merits between one and the other is beyond the technical and philosophic scope of this paper. At our institution, the majority of fusion procedures > 3 levels are instrumented freehanded. The decision to use navigated assistance for redirection has emerged from (1) ease of synced workflow with an intraoperative CT scan and (2) belief in the benefit of visual-tactile synchrony gained with robotic assisted recannulation of a new tract. In essence, navigation is our “safety check” to complement other approaches when ensuring an accurate new cannulation corridor.

Our results suggest the ongoing possibility of hybrid approaches between shared-control systems and skilled spine surgeons. We demonstrate the use of the navigation as safety check for accurate screw redirection, reporting no negative clinically outcomes or return to the OR and 97.8% accuracy based on post-redirection radiographic findings. There were no aberrancies in neuromonitoring. We also address navigation for non-pedicular approaches for iliac fixation with 100% accuracy, which has limited evaluation in the literature.

Limitations of our study include lack of post-operative CT for true axial comparison of screw redirection. We sought to overcome this limitation via multimodal surrogates for accurate screw placement, including post-operative exam, neuromonitoring, and independent neuroradiologic evaluation of pre- and post-fluoroscopic and X-ray images of redirected screws. This study is also a retrospective case series without matched control and cannot be used to extrapolate comment regarding operative time or incidence of poor outcome in patients without navigated redirection.

## Conclusions

Redirection of suboptimally placed thoracolumbar and sacroiliac screws can be performed using intraoperative O-Arm and navigation as a means to detect and directly visualize appropriate placement. We report an overall satisfactory redirection of 97.8% screws in post-operative imaging and no return to the OR for revision. Additional studies are required to compare operative time between navigated and non-navigated screws.

## Data Availability

Not appplicable.
